# Supplementary material for: Studies on the Reactions of Biapenem with VIM Metallo β-Lactamases and the Serine β-Lactamase KPC-2
Source: Antibiotics (Basel). 2022 Mar 16;11(3):396. doi: 10.3390/antibiotics11030396 (PMC8944426; doi:10.3390/antibiotics11030396)
Supplement: Supplementary file 1 [file antibiotics-11-00396-s001.zip › antibiotics-1591213-supplementary.pdf]

**Studies on the Reactions of Biapenem with VIM metallo β-lactamases and the serine β-lactamase KPC-2**

**Supporting Information**

Anka Lucic[1], Tika R. Malla[1], Karina Calvopiña[2], Catherine L. Tooke[2], Jürgen Brem[1], Michael A. McDonough[1], James Spencer[1] and Christopher J. Schofield[1,*]

[1] Chemistry Research Laboratory, The Department of Chemistry and the Ineos Oxford Institute for Antimicrobial Research, University of Oxford, 12 Mansfield Road, Oxford OX1 3TA, UK,
[2] Biomedical Sciences Building, School of Cellular and Molecular Medicine, Faculty of Life Sciences, University of Bristol, University Walk, Bristol BS8 1TD, UK,

* Correspondence: christopher.schofield@chem.ox.ac.uk

# Table of Contents


***Studies on the Reactions of Biapenem with VIM metallo β-lactamases and the serine-β-lactamase KPC-2*** ........ ***1***

***Supporting Information*** ........ ***1***

**Table S1. Chemical shift assignments for the major biapenem-derived hydrolysis product** ........ 3
**Table S2. Data collection and refinement statistics**. ........ 4
**Figure S1. $^1$H NMR time course analysis of methyl group resonances of species formed on reaction of biapenem with KPC-2** ........ 5
**Figure S2. $^1$H NMR time course analysis of methyl group resonances of species formed on reaction of biapenem with VIM-1** ........ 6
**Figure S3. $^1$H NMR time course analysis of methyl group resonances of products formed on reaction of biapenem with VIM-2** ........ 7
**Figure S4. $^1$H-NMR time course analyses for reaction of biapenem with VIM-1 and VIM-2 (MBLs) and KPC-2 (an SBL)** ........ 8
**Figure S5. View of the overall fold of the VIM-2: biapenem derived complex** ........ 9
**Figure S6 (A) Interactions between the biapenem derived enamine ligand (3) and the VIM-2 active site. (B) Interactions between the biapenem derived (2*S*)-imine (1) derived ligand and the VIM-2 active site** ........ 9
**Figure S7. Comparisons of conformations of carbapenem and penem derived ligands with VIM-1 and VIM-2.** ........ 10
**Figure S8. Overlay of the conformations of biapenem derived enamine and imine ligands with β-lactamases and penicillin binding proteins** ........ 11

| Position | $^{13}C$ (ppm) | $^{1}H$ (ppm) |
|---|---|---|
| **1** | 43.97 | 2.56 |
| **2** | 57.33 | 3.68 |
| **3** | | |
| **4** | | |
| **5** | 72.8 | 4.35 |
| **6** | 55.92 | 2.6 |
| **7** | 180.18 | |
| **8** | 68.11 | 4.03 |
| **9** | 20.75 | 1.19 |
| **10** | 172.45 | |
| **11** | 13.49 | 1.01 |
| **12,14** | 53.66 | 4.57,5.04 |
| **13** | | |
| **15,16** | 142.84 | 8.93 |

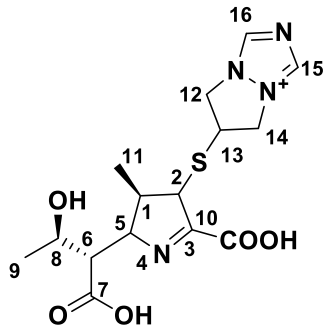


***Table S1****. Chemical shift assignments for the major biapenem-derived hydrolysis product.* The chemical shifts and the C2 coupling constants (J 3.6, 1.3 Hz) of this hydrolysis product suggest that it is likely the (2*S*)-imine based on previous studies [8,9].

| | VIM-2: biapenem |
|---|---|
| **PDB accession** | 6Y6J |
| **Data collection** | |
| **Facility/Beamline** | Diamond I04-1 |
| Space group | *I*222 |
| Molecules/ASU | 1 |
| Cell dimensions | |
| a, b, c (Å) | 64.324, 74.02, 78.354 |
| α, β, γ (°) | 90.0, 90.0, 90.0 |
| Wavelength (λ) | 0.9159 |
| Resolution (Å) | 27.61-1.33 (1.36-1.33) |
| $R_{pim}$ | 0.030 (0.205) |
| CC 1/2 | 0.998 (0.872) |
| I / σI | 14.6 (3.0) |
| Completeness (%) | 99.56 (98.96) |
| Multiplicity | 13.3 (12.6) |
| | |
| **Refinement** | |
| Resolution (Å) | 27.61-1.5 |
| No. reflections | 30179 |
| $R_{work}$ / $R_{free}$ | 0.1287 / 0.1573 |
| No. non-H atoms | 2048 |
| Protein | 1792 |
| Solvent | 200 |
| Zn | 3 |
| Ligands | 56 |
| Avg. B-factors (Å$^2$) | |
| Protein | 17.40 |
| Solvent | 31.03 |
| Ligands | 16.49 |
| R.m.s. deviations | |
| Bond lengths (Å) | 0.009 |
| Bond angles (°) | 1.15 |
| Ramachandran (%) | |
| Outliers | 0.00 |
| Favoured | 98.25 |

**Table S2. Data collection and refinement statistics**. High resolution statistics (1.36-1.33 Å) are in parentheses. Due to poor completeness, the resolution for data processing was cut of to 1.5 Å, with a high resolution shell of 1.556-1.5 Å.

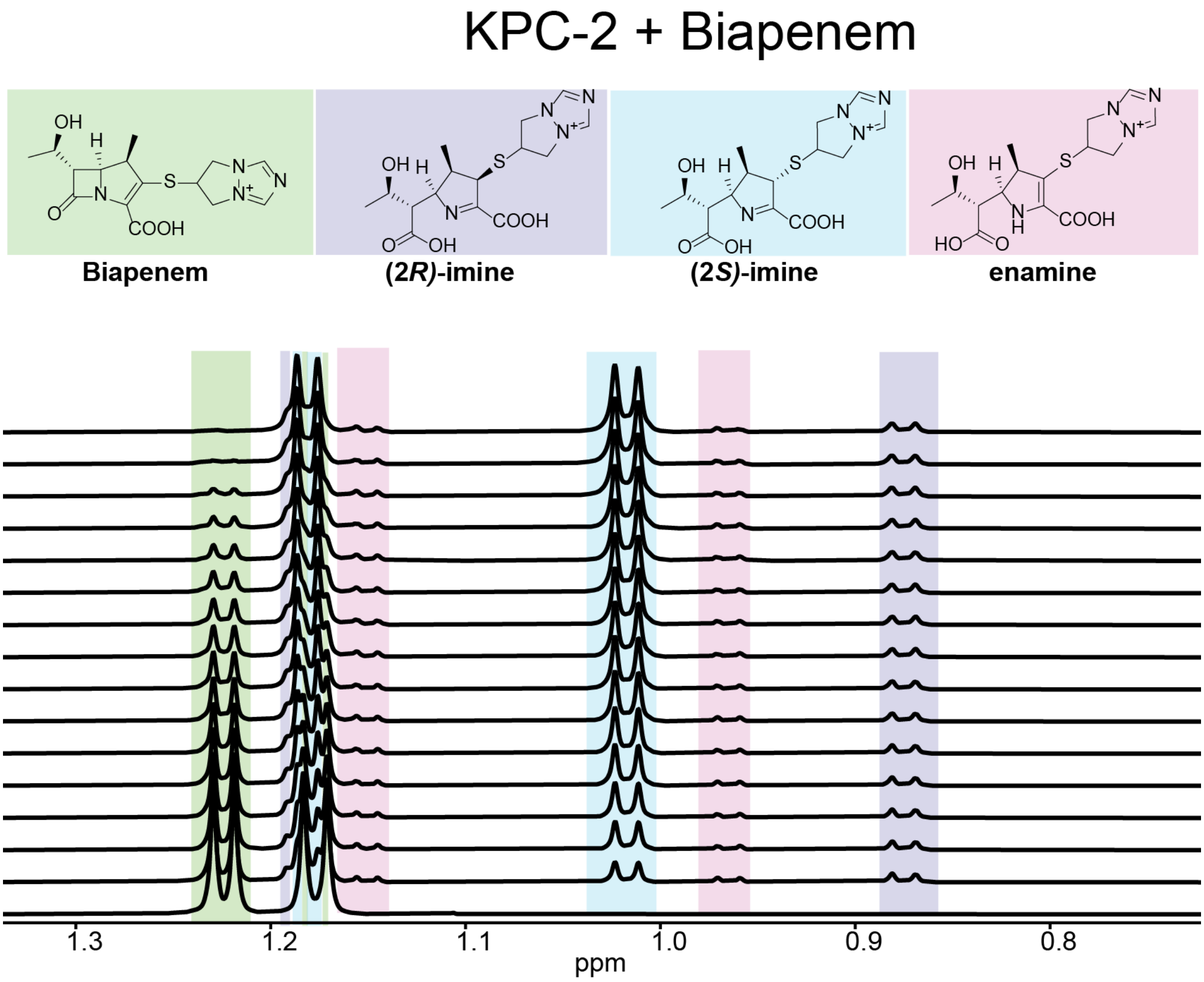


***Figure* S*1*. 1H NMR time course analysis of methyl group resonances of species formed on reaction of biapenem with KPC-2.** Biapenem (5 mM, green) was treated with KPC-2 (280 nM) in 50 mM sodium phosphate at pH 7.5, 10% (v/v) $D_2O$. Evidence was accrued for formation of (2*R*)-imine, (2*S*)-imine and enamine products (blue, purple, and pink, respectively). 15 spectra were acquired (16 scans, every 2.5 minutes) over 37.5 minutes (stacked from bottom / first spectrum to top). The bottom spectrum is of biapenem (5 mM) without added enzyme.

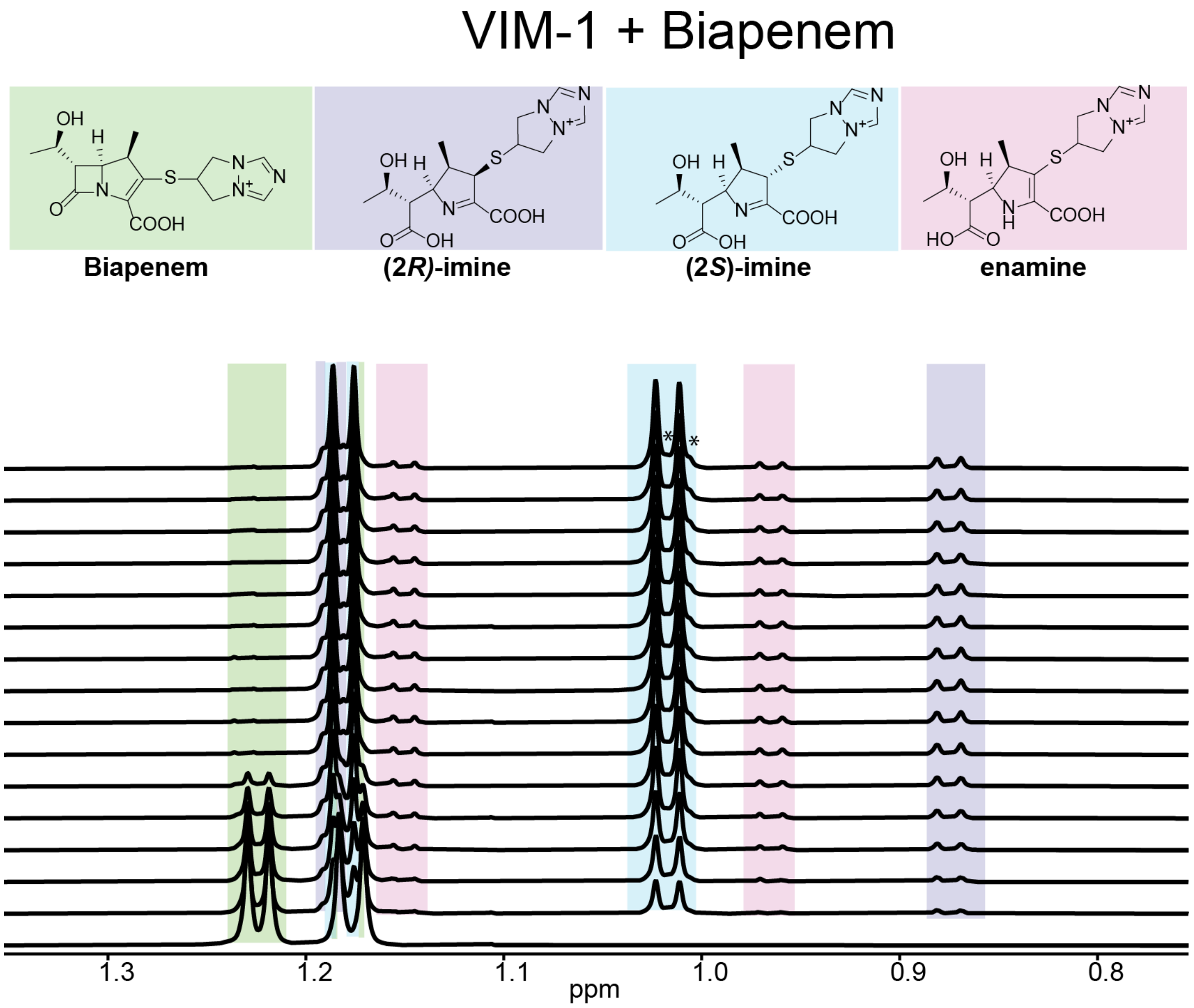


*Figure S2.* **1H NMR time course analysis of methyl group resonances of species formed on reaction of biapenem with VIM-1**. Biapenem (5 mM, green) was treated with VIM-1 (280 nM) in 50 mM sodium phosphate at pH 7.5, 10% (v/v) $D_2O$. Unassigned product peaks are labelled with asterisks. Evidence was accrued for formation of (2*R*)-imine, (2*S*)-imine and enamine products (blue, purple, and pink, respectively). 15 spectra were acquired (16 scans, every 2.5 minutes) over 37.5 minutes (stacked from bottom / first spectrum to top). The bottom spectrum is biapenem (5 mM) without added enzyme.

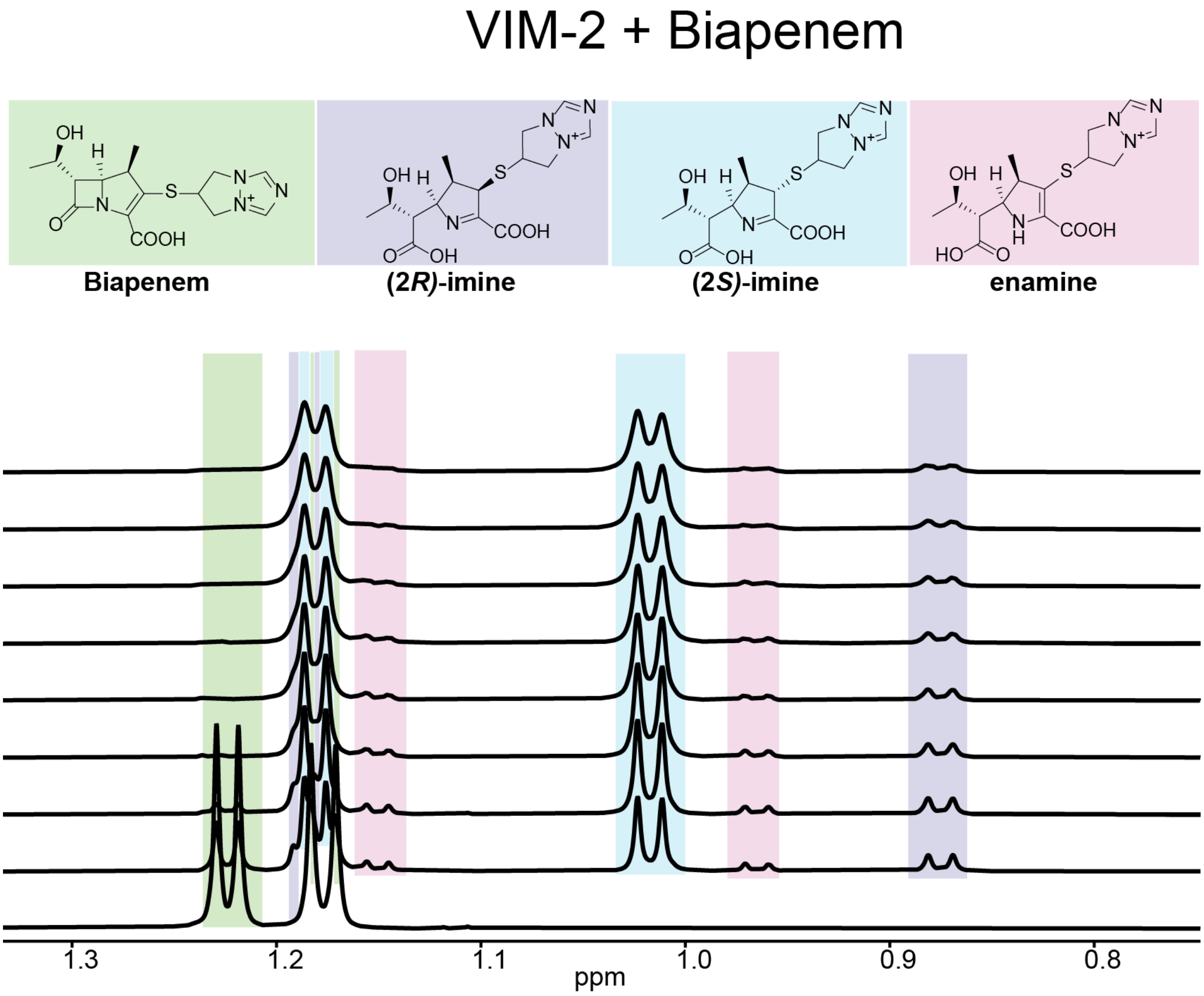


***Figure S3*. $^{1}$H NMR time course analysis of methyl group resonances of products formed on reaction of biapenem with VIM-2**. Biapenem (5 mM, green) was treated with VIM-2 (280 nM) in 50 mM sodium phosphate at pH 7.5, 10% (v/v) $D_2O$. Evidence was accrued for formation of (2*R*)-imine and (2*S*)-imine and enamine products (blue, purple, and pink, respectively). 8 spectra were acquired (16 scans, every 2.5 minutes) over 20 minutes (stacked from bottom / first spectrum to top). The bottom spectrum is biapenem (5 mM) without added enzyme.

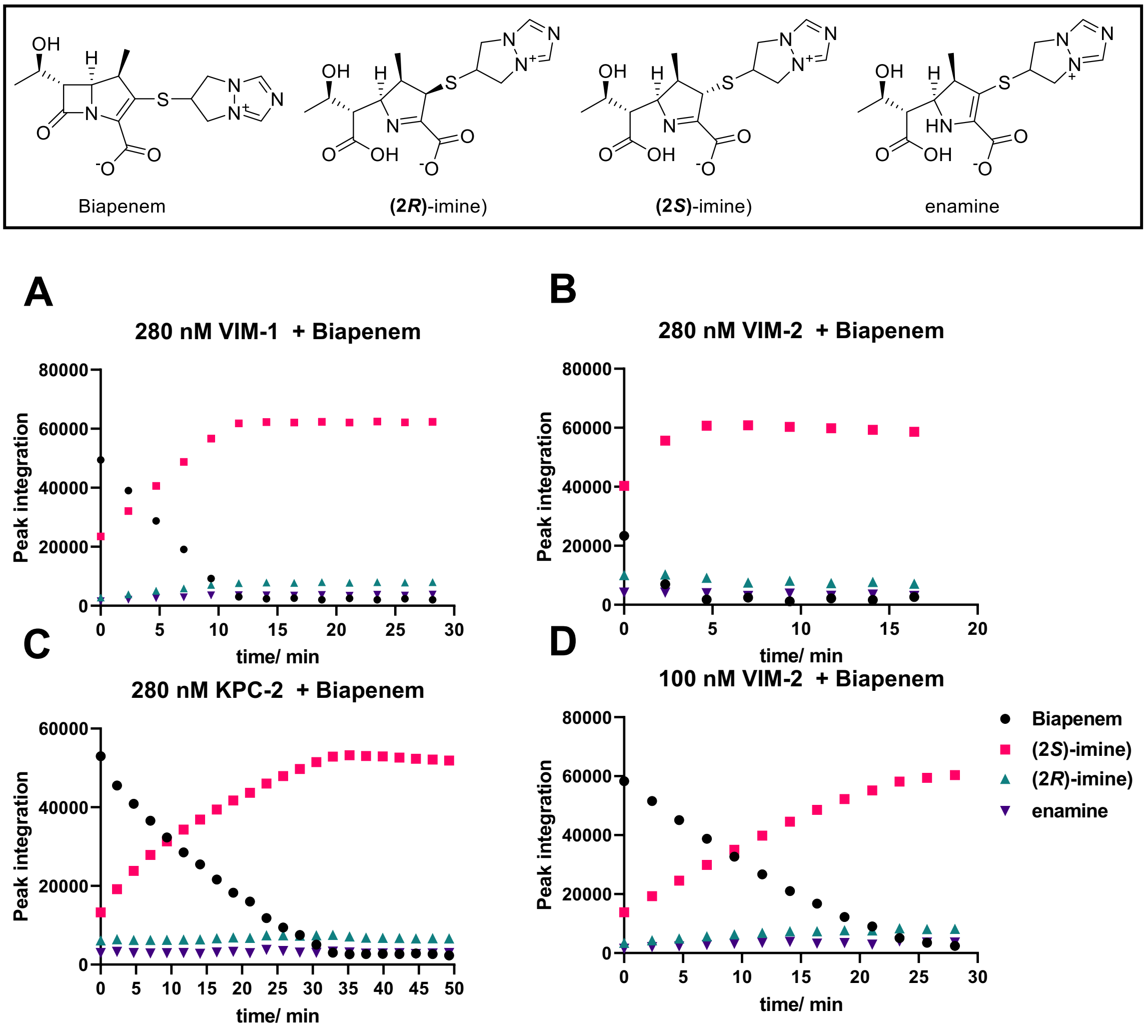


*Figure S4* **1H-NMR time course analyses for reaction of biapenem with VIM-1 and VIM-2 (MBLs) and KPC-2 (an SBL).** Methyl group resonance peak integrations from time course data were analysed to monitor reaction of biapenem to give (2*R*)-imine, (2*S*)-imine and enamine (blue, pink, and purple, respectively) products using a Bruker AVIIIHD 600 MHz machine. Biapenem (5 mM) was treated with (**A**) VIM-1 (280 nM), (**B**) VIM-2 (280 nM), (**C**) KPC-2 (280 nM) and (**D**) VIM-2 (100 nM) in 50 mM sodium phosphate at pH 7.5, 10% (v/v) $D_2O$. Integration of methyl resonance of biapenem was normalised ((integration of biapenem) / (integration of biapenem + (2*R*)-imine + (2*S*)-imine + enamine) *100 was used to calculate the percentage of biapenem turnover.

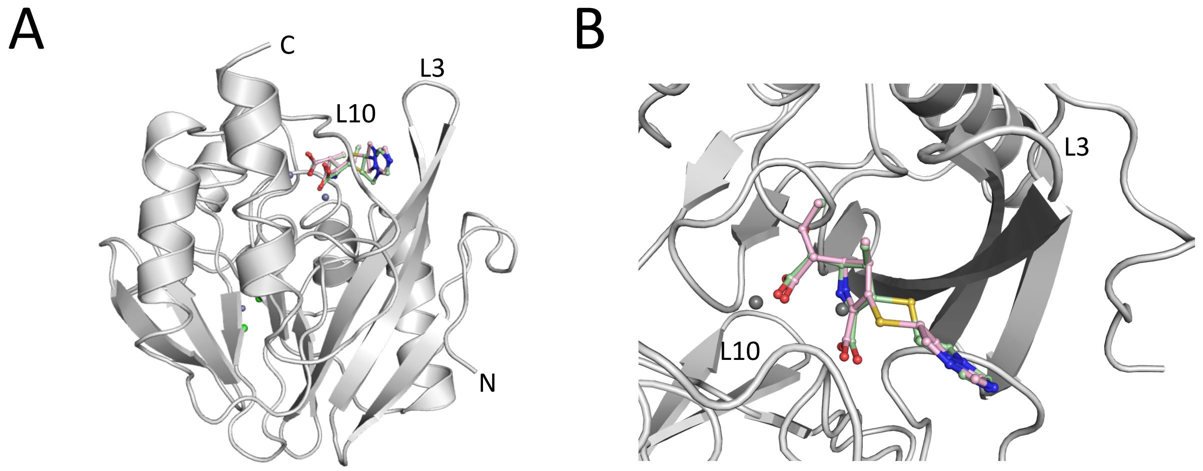


**Figure S5. View of the overall fold of the VIM-2: biapenem derived complex.** A) The structure was processed in the space group *I*222 (PDB 6Y6J). Zinc ions are shown as grey spheres and chloride ions are in green. B) Active site view of VIM-2 complexed with biapenem derived products. Relevant loops for interaction are labelled. The biapenem derived enamine ligand is in pale pink, and the biapenem derived imine ligand is in pale green (Figure S6).

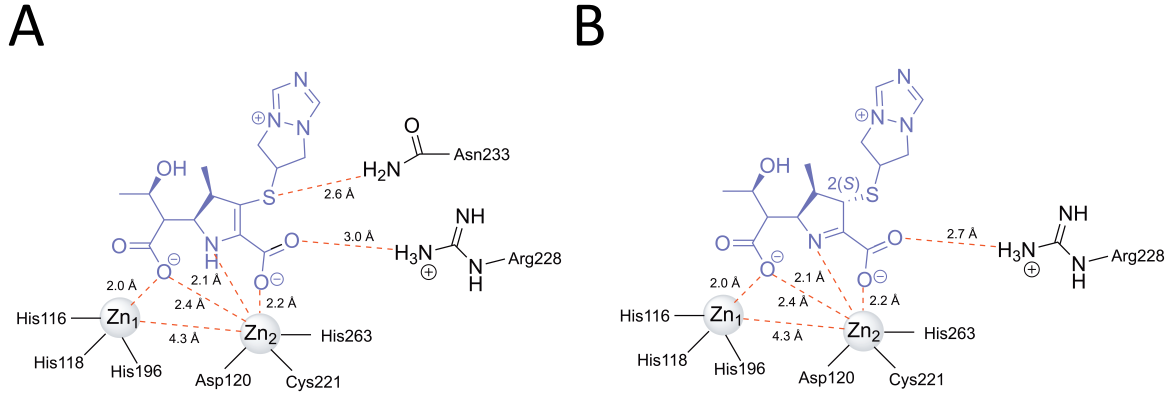


**Figure S6.** (A) Interactions between the biapenem derived enamine ligand and the VIM-2 active site. (B) Interactions between the biapenem derived (2S)-imine derived ligand and the VIM-2 active site**.**

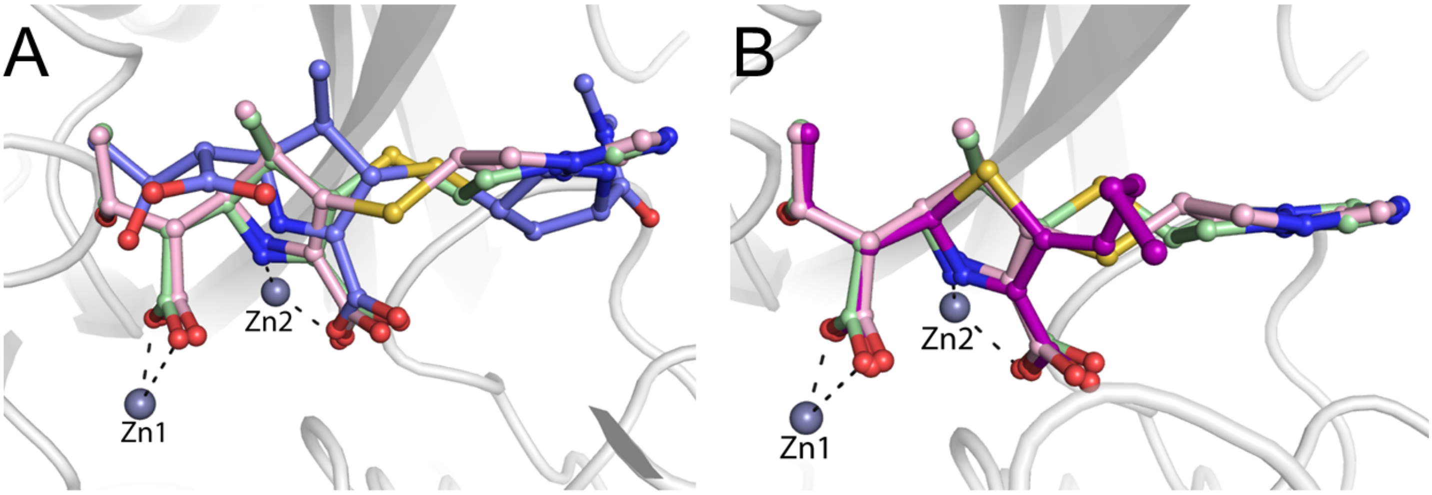


**Figure S7. Comparisons of conformations of carbapenem and penem derived ligands with VIM-1 and VIM-2.** (**A**) View of VIM-1 complexed with (i) the meropenem derived (2*S*)-imine ligand in slate blue (PDB 5N5I [19]); (ii) the VIM-2 biapenem derived (2*S*)-imine in green; and (iii) the VIM-2 biapenem derived enamine in pink. (PDBs: 6Y6J). (**B**) View of VIM-2 complexed with the (i) faropenem derived product (purple) (PDBs: 7A5Z [20]); (ii) the biapenem derived (2*S*)-imine in green and (iii) the biapenem derived enamine in pink. (PDBs: 6Y6J). Note that the binding modes of the faropenem derived imine (Z)-alkene (dark purple) and biapenem (2*S*)-imine derived ligands are similar.

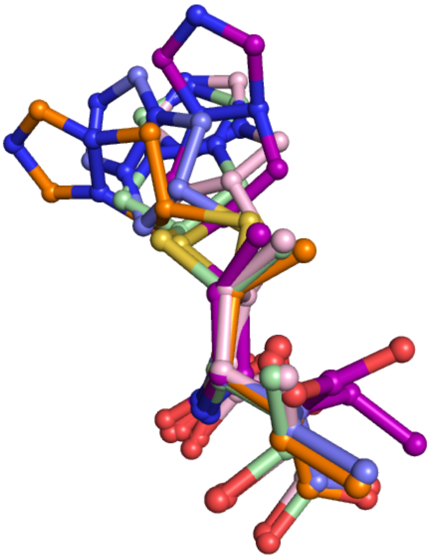

**Figure S8. Overlay of the conformations of biapenem derived enamine and imine ligands with β-lactamases and penicillin binding proteins.** Colour code: VIM-2 (class B1 MBL) complexed with the biapenem derived enamine - light pink; VIM-2 complexed with the biapenem derived (2*S*)-imine - light green (PDB 6Y6J); PBP2X complexed with the biapenem derived enamine - slate blue (PDB 2ZC3), PBP1A complexed with the biapenem substrate - orange) (PDB 2ZC5) [24], and CphA (class B2 MBL) complexed with the biapenem derived enamine - purple (PDB 1X8I)[23].
